# Prevalence of HIV-1 Drug Resistance among Women Screening for HIV Prevention Trials in KwaZulu-Natal, South Africa (MTN-009)

**DOI:** 10.1371/journal.pone.0059787

**Published:** 2013-04-09

**Authors:** Urvi M. Parikh, Photini Kiepiela, Shayhana Ganesh, Kailazarid Gomez, Stephanie Horn, Krista Eskay, Cliff Kelly, Barbara Mensch, Pamina Gorbach, Lydia Soto-Torres, Gita Ramjee, John W. Mellors, on behalf of the IPTc Taskforce

**Affiliations:** 1 Department of Infectious Diseases, University of Pittsburgh School of Medicine, Pittsburgh, Pennsylvania, United States of America; 2 HIV Prevention Research Unit, Medical Research Council, Durban, South Africa; 3 FHI 360, Research Triangle Park, North Carolina, United States of America; 4 Statistical Center for HIV/AIDS Research and Prevention, Fred Hutchinson Cancer Research Center, Seattle, Washington, United States of America; 5 Population Council, New York, New York, United States of America; 6 Department of Epidemiology, University of California Los Angeles, Los Angeles, California, United States of America; 7 Division of AIDS, National Institute of Allergy and Infectious Diseases, National Institutes of Health, Bethesda, Maryland, United States of America; Wits Reproductive Health and HIV Institute, South Africa

## Abstract

**Background:**

A major concern with using antiretroviral (ARV)-based products for HIV prevention is the potential spread of drug resistance, particularly from individuals who are HIV-infected but unaware of their status. Limited data exist on the prevalence of HIV infection or drug resistance among potential users of ARV-based prevention products.

**Methods:**

A cross-sectional study of reproductive-aged women who presented to screen for an HIV prevention trial was conducted at 7 clinical sites in Durban, South Africa. CD4+T cell counts, HIV-1 RNA levels and population sequencing of the protease and reverse transcriptase genes were performed for all women with 2 positive HIV rapid tests. Resistance mutations were identified using the Stanford Calibrated Population Resistance Tool.

**Results:**

Of the 1073 evaluable women, 400(37%) were confirmed as HIV-infected. Of those, plasma HIV-1 RNA was detectable in 365/400(91%) and undetectable(<40 copies/ml) in 35/400(9%) women. 156 women(39%) were eligible for antiretroviral therapy (CD4+T cell counts<350 cells/mm^3^) and 50(13%) met criteria for AIDS(CD4<200 cells/mm^3^). Of 352 plasma samples(>200 copies/ml) analyzed for drug resistance, 26(7.4%) had nucleoside reverse transcriptase inhibitor (NRTI), non-nucleoside reverse transcriptase inhibitor (NNRTI) or protease inhibitor (PI) drug resistance mutations. Among those with resistance, 18/26 participants(62%) had single-class NNRTI resistance and 5/26(19%) had dual-class NRTI/NNRTI. Major mutations in reverse transcriptase included K65R(n = 1), L74I(n = 1), K103N(n = 19), V106M(n = 4), Y181C(n = 2), M184V(n = 4), and K219E/R(n = 2). Major PI-resistance mutations were rare: M46L(n = 1) and I85V(n = 1). All participants were infected with subtype C virus, except one infected with subtype A.

**Conclusions:**

In women from Durban, South Africa screening for an HIV prevention trial, the HIV prevalence was high (37%) and HIV drug resistance prevalence was above 5%. This study highlights the potential challenges faced when implementing an ARV-based prevention product that overlaps with first-line antiretroviral therapy. Effective screening to exclude HIV infection among women interested in uptake of ARV-based HIV prevention will be essential in limiting the spread of ARV resistance.

## Introduction

Women are disproportionately burdened by human immunodeficiency virus (HIV) infection, particularly in sub-Saharan Africa, where approximately three-quarters of new HIV-1 infections are in young women aged 15–24 years [1], [2]. Recent clinical trials evaluating tenofovir as a potential chemo-preventative agent have screened thousands of women for participation in large-scale studies including FEM-PrEP, CAPRISA-004, TDF2 and MTN-003 (VOICE) [3]. Inevitably, some women who present to the clinic intending to participate in an HIV-prevention trial discover they are HIV positive or already have knowledge of their status but still seek HIV prevention products or trial participation for other reasons [4]. This group of women is critical to understand both from a virologic and behavioral perspective because the future success and large scale implementation of an ARV product for HIV prevention largely depends on targeting the appropriate population for its use.

One of the major concerns of using ARV-based products for HIV prevention is the potential for drug resistance, particularly in individuals who are HIV infected and unaware of their status. In a survey of 5821 women and men from 16 rural communities in KwaZulu-Natal South Africa, 68% reported they had never been tested for HIV [5]. A recent modeling analysis identified inadvertent PrEP use by already-infected individuals as having the greatest influence on the potential for emergence and spread of resistance arising from PrEP rollout [6]. To date, the 5 cases of resistance that have occurred in a total of 172 seroconverters from the use of tenofovir-based pre-exposure prophylaxis (PrEP) have been from participants on active antiretroviral (ARV) arms who enrolled during the acute phase of infection: 0/35 in the TFV gel arm in CAPRISA-004 [7]; 2/36 in the oral TDF-FTC arm in iPrEX [8], 1/9 in the TDF2 study [9], and 2/92 from the Partners in PrEP serodiscordant couple study, where 1 case occurred in the TDF arm, and 1 case occurred in the TDF-FTC arm [10].

Transmitted resistance in the general population could also potentially compromise the success of ARV-based prevention if circulating variants are resistant to the products used for topical or oral agents. While most studies conducted in sub-Saharan Africa thus far have identified low rates of transmitted ARV resistance, mathematical modeling and experience from resource-rich countries suggest that once antiretroviral therapy (ART) coverage increases, the rate may rise [11], [12]. In South Africa, the frequency of transmitted resistance has been variable: 1.1% in Pretoria, 4.5% in Johannesburg, 4.8% in White River and as high as 9.3% in Northeastern South Africa [13], [14]. An analysis of 1690 sequences from recent seroconverters in KwaZulu-Natal reported the prevalence of resistance as <5% [15]. Recent surveys using the WHO threshold surveillance method of treatment-naïve and/or recently diagnosed pregnant women from antenatal clinics in KwaZulu-Natal have reported low resistance prevalence of <5% but increasing to 5–15% for NNRTIs [16], [17].

The objective of MTN-009 was to provide a current estimate of the prevalence of ARV resistance in a subset of women screening to participate in HIV prevention trials.

## Methods

### Design

**Figure 1:**
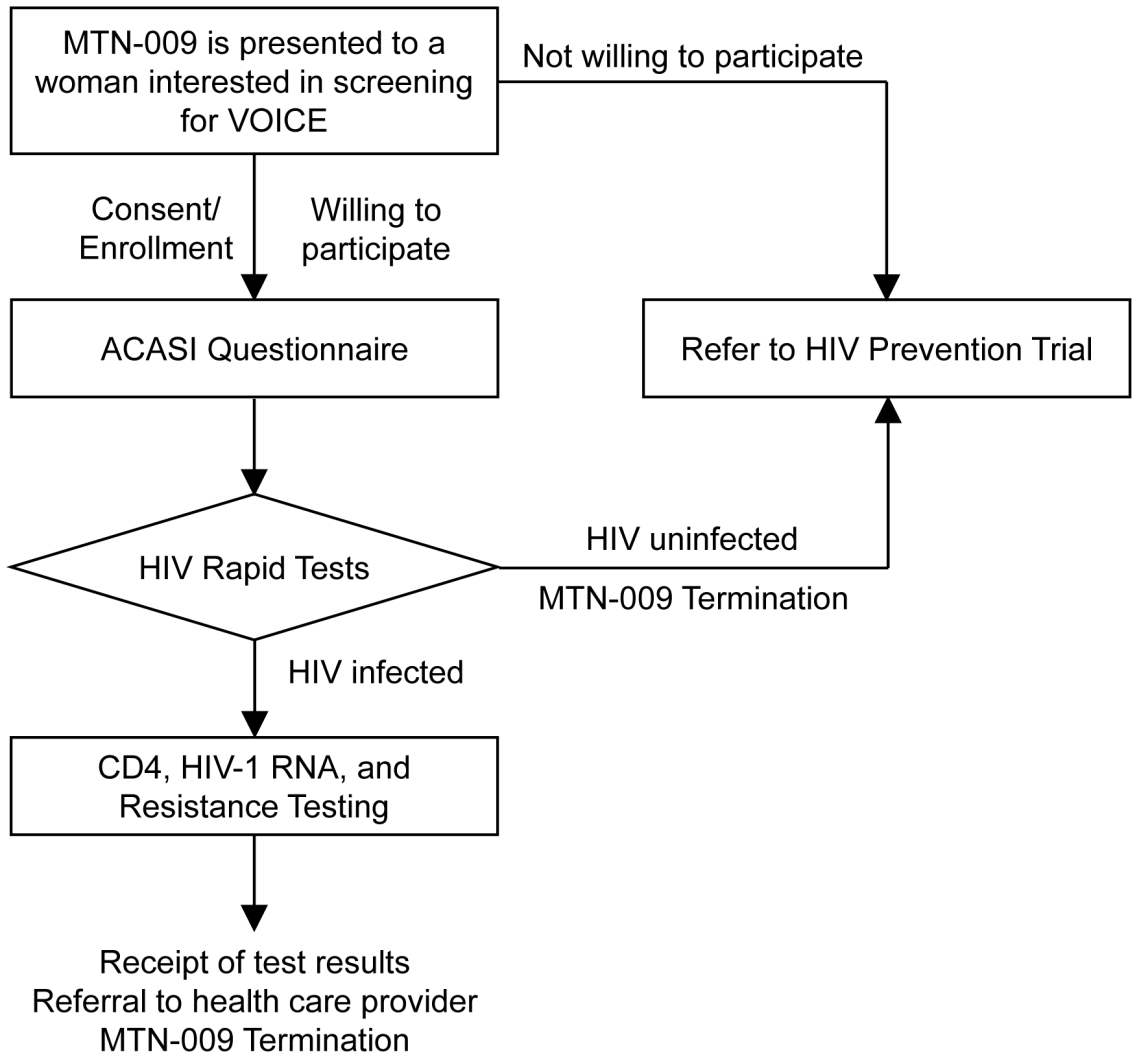
MTN-009 Study Schema.

MTN-009 was a cross-sectional study conducted at seven sites of the HIV Prevention Research Unit, Medical Research Council between September 2010 and March 2011. Clinical sites are located in semi-rural and urban areas in the greater Durban area of KwaZulu-Natal. These include Botha’s Hill, Chatsworth, Isipingo, Overport, Tongaat, Umkomaas, and Verulam. Participants were not specifically recruited for MTN-009, but interest in participation in this study was sought among those who presented to the study site to screen for the VOICE/MTN-003 trial and whose HIV status was unknown. VOICE is a phase 2B safety and effectiveness study of tenofovir 1% gel, tenofovir disoproxil fumarate (TDF) tablet and emtricitabine/TDF tablet for the prevention of HIV infection in women (http://www.mtnstopshiv.org/studies/70). The target sample size for MTN-009 was 1000 women aged 18–40 years to enroll 350 HIV positive evaluable participants. As shown in Figure 1, after obtaining informed consent, women enrolled in MTN-009 and underwent an audio computer assisted self-interview questionnaire (ACASI) before rapid testing for HIV to determine HIV status by serology. All participants were counseled, and those with two negative rapid tests were referred to the VOICE study, while those who were seropositive underwent additional laboratory testing described below. The protocol, informed consent forms, and all study materials were reviewed and approved by the National Institute of Allergy and Infectious Diseases Division of AIDS (NIAID/DAIDS) in the United States, and by the Medical Research Council Ethics Committee in South Africa. The MTN-009 study was registered at www.Clinical-Trials.gov (NCT01204814) and the protocol can be found at http://www.mtnstopshiv.org. All participants provided written informed consent to participate in this study.

### Laboratory Methods

Two simultaneously performed HIV rapid tests, at least one of which was FDA-approved (Determine, Abbott Laboratories, Johannesburg, SA; Unigold, Trinity Biotech, Wicklow, Ireland; or Oraquick, OraSure Technologies, Bethlehem, PA) were used to identify MTN-009 participants as HIV-1 seropositive. Infection was confirmed by the Bio-Rad GS HIV-1/2+O Enzyme Immunoassay (Hercules, CA). In addition, Bio-Rad GS HIV-1 Western blot was used to confirm infection status for participants with either discordant rapids or with dual positive rapids and undetectable HIV-1 RNA (<40 copies/ml). Plasma HIV-1 RNA levels (with a lower limit of detection of 40 copies/ml) using the *m*2000 RealTi*m*e HIV-1 system (Abbott Laboratories, Abbott Park, IL) and CD4+ T cell counts using the FACS calibur (BD diagnostics, Woodmead, Gauteng, South Africa) were determined for all HIV positive participants. Plasma HIV-1 genotyping was attempted for all participants with RNA levels >200 copies/ml using the ViroSeq 2.0 Genotyping Method (Celera, Alameda, CA) which targets sequencing of the entire *protease* gene from amino acids 1–99 and two thirds of the *reverse transcriptase* gene from amino acids 1–335. The 36 samples that did not amplify with FDA-approved primers provided with the ViroSeq kit, or that did not successfully sequence with at least 5 bi-directional overlapping primers were re-amplified and/or re-sequenced using in-house primers provided by Celera. RNA from samples with low viral RNA levels (200–1000 copies/ml) were concentrated before performing RT-PCR. All sequences were manually edited and nucleotide positions with multiple peaks present at greater than 20% above background were considered mixtures. Resistance mutations were identified using the Stanford Calibrated Population Resistance Tool [18] and samples were considered to be resistant if they contained one or more mutations as defined by the Bennett WHO list of transmitted resistance [19]. Samples were subtyped using the Rega Subtyping tool. Participants with CD4+ T cell counts of <200 cells/mm^3^ and resistance mutations were counseled on what the test measured and what the results signify.

### Statistical Methods

Descriptive statistics were used to summarize characteristics of the cohort and the prevalence and types of resistance mutations. Specifically, the number and percentage in each category were determined for categorical variables, and the mean, median, standard deviation, quartiles and range (minimum, maximum) were calculated for continuous variables. The exact 95% confidence interval for the prevalence of resistance was calculated using the Clopper-Pearson method for a binomial distribution.

## Results

### Study Population

**Figure 2:**
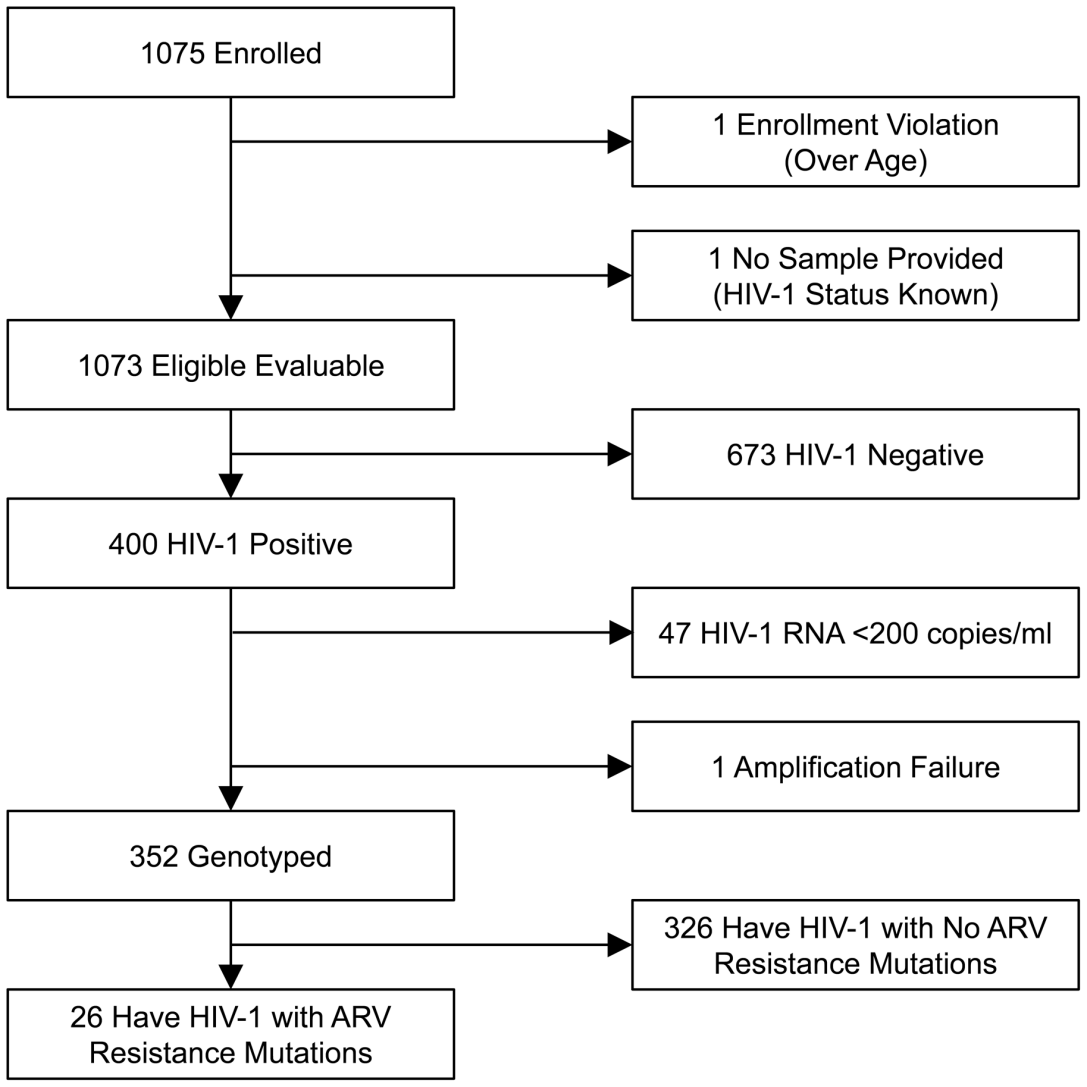
Consort Diagram.

1075 women who presented to screen for VOICE were offered participation and subsequently enrolled in MTN-009; no woman declined enrollment. Two participants were excluded due to enrollment violations: 1) above age 40, and 2) revealed prior knowledge of HIV positive status after enrollment (Figure 2). Of the remaining 1073 participants, the mean age was 25.6 years, and predominant race was Zulu (87%). Almost all participants (1015 [99%]) reported having a primary sex partner but only 43 (4%) reported being married. Most participants earned their own income (70%) and had at least some secondary school education (92%). The mean number of children born to participants was 1.4±1.1 (Table 1). More than a third of participants (400/1073) were confirmed to be HIV-1 positive, resulting in a prevalence of 37% in the MTN-009 study population.

**Table 1:** Demographic Factors for All Evaluable Participants and the Subset that was HIV Positive and Tested for Drug Resistance in MTN-009.

Participant Factors	All Evaluable Participants (N = 1073)	HIV+ & tested for resistance (N = 352)
Mean age, years (SD)	25.6 (5.6)	26.8 (5.4)
Married	43 (4%)	11 (3%)
Has a primary sex partner	1015 (99%)	335 (98%)
Living with husband/primary sex partner	200 (19%)	63 (18%)
Earns her own income	747 (70%)	243 (69%)
Has at least some secondary school education	987 (92%)	315 (89%)
Mean (SD) number of children given birth to	1.4 (1.1)	1.4 (1.1)
Race		
Zulu	931 (87%)	300 (85%)
Xhosa	111 (10%)	45 (13%)
Indian	12 (1%)	0 (0%)
Other	17 (2%)	7 (2%)

Notes: SD = Standard Deviation.

### CD4+ T Cell Counts and HIV-1 RNA Levels

**Figure 3:**
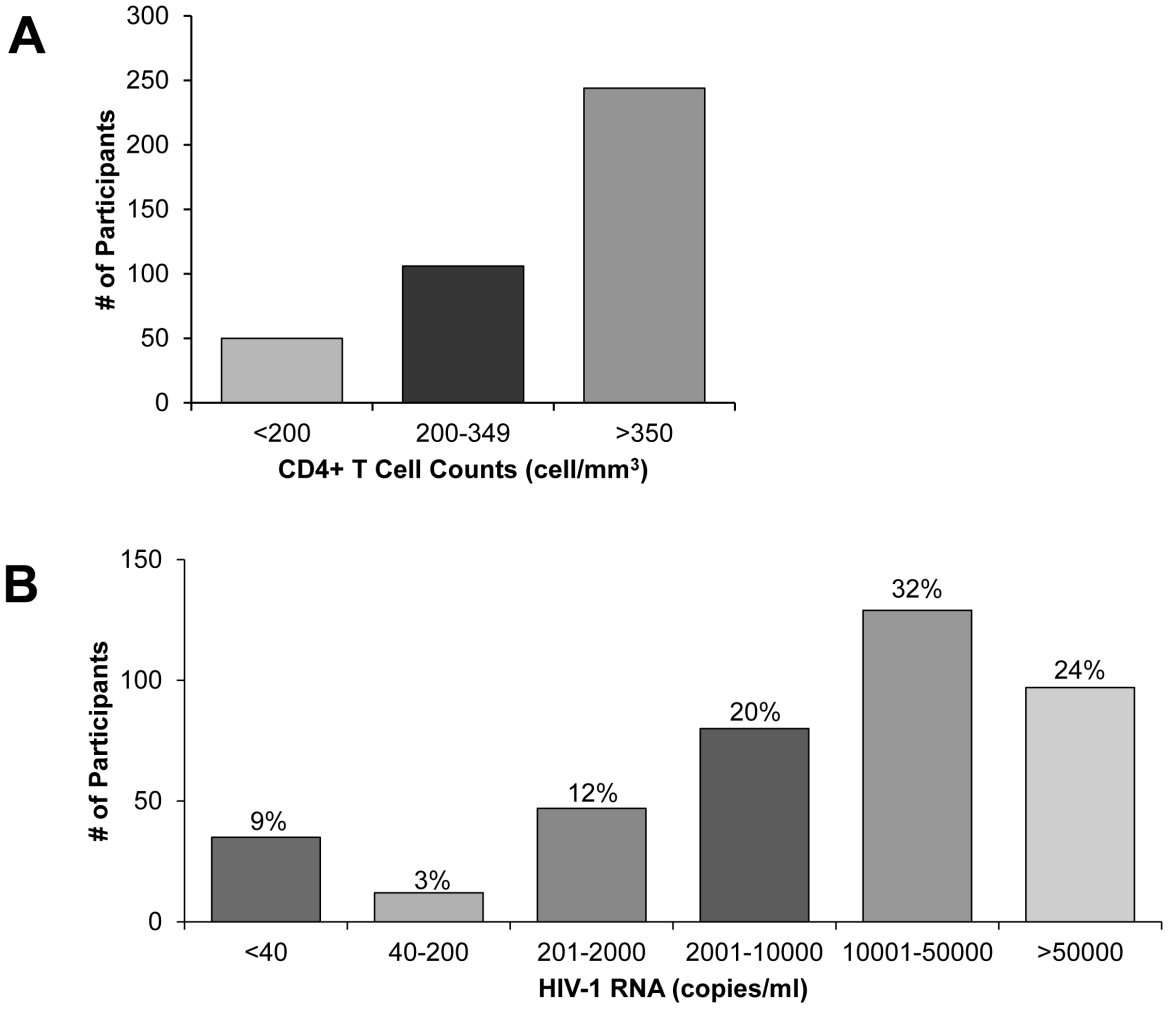
CD4+ T Cell Counts and HIV-1 RNA Levels of HIV-1 Positive Participants. Histogram showing frequency of HIV positive participants (n = 400) that have (A) CD4+ T cell counts <200 cells/mm^3^ indicating risk for AIDS, 200–349 cells/mm^3^ indicating treatment eligibility, and >350 cells/mm^3^; and (B) varying levels of HIV-1 RNA (copies/ml).

Of 400 HIV-1 positive participants, 244 (61%) had CD4+ T cell counts above 350 cells/mm^3^ (Figure 3). 156 (39%) had CD4 counts below 350 cells/mm^3^ indicating eligibility for antiretroviral treatment if pregnant, co-infected with tuberculosis or in WHO clinical state IV according to the South African guidelines at the time period during which the study took place (September 2010– March 2011) [20]. Fifty women (13%) had CD4 counts below 200 cells/mm^3^. These participants met ART eligibility in South Africa irrespective of clinical stage [20] and are considered to have AIDS according to the Center for Disease Control and Prevention’s AIDS surveillance case definition [21].

Plasma HIV-1 RNA levels ranged from undetectable (<40) to 2,674,072 copies/ml. Excluding those with undetectable values, mean and median HIV-1 RNA copies/ml were 75,449 and 18,924 copies/ml respectively. Plasma HIV-1 RNA copies/ml were below detectable range in 35/400 (8.8%) of participants, and an additional 12 participants (3%) had detectable HIV-1 RNA levels that were at or below 200 copies/ml (Figure 3).

### Antiretroviral Resistance

**Figure 4:**
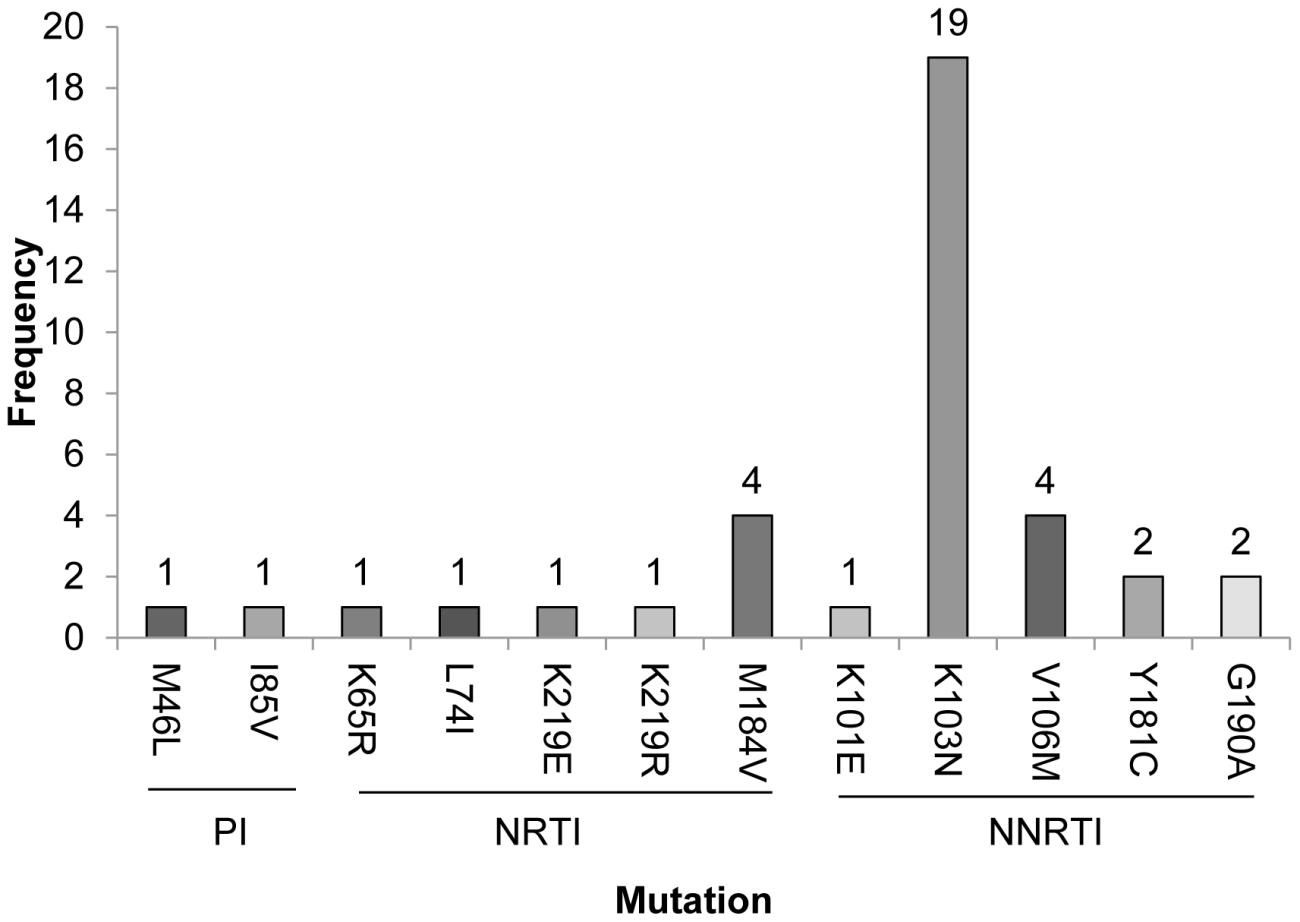
Frequency of ARV Resistance Mutations. Histogram showing number of women with drug resistant HIV infection that had each of the following protease inhibitor (PI), nucleoside reverse transcriptase inhibitor (NRTI) or non-nucleoside reverse transcriptase inhibitor (NNRTI) mutations M46L, I85V, K65R, L74I, K219E, M184V, K101E, V106M, Y181C or G190A. Resistance mutations were identified using the Stanford Calibrated Population Resistance Tool.

Most participants (352/400 [88%]) had HIV-1 RNA levels above 200 copies/ml and plasma virus from all but one was successfully sequenced for determination of HIV resistance using standard, commercially-available genotyping methods to identify mutations in the *reverse transcriptase* and *protease* genes in the HIV-1 polymerase region. The majority of samples (326 [92.6%]) had HIV-1 with no known ARV resistance mutations, whereas 26 (7.4%; 95% CI [4.9, 10.6]) had at least one known ARV-resistance mutation, as defined by the World Health Organization (WHO) list of transmitted drug resistance [19] (Figure 4). K103N was the most frequently detected mutation, present in 19/26 samples having drug resistance (73%). There were also multiple occurrences of V106M (n = 4), M184V (n = 4), Y181C (n = 2) and G190A (n = 2). The following mutations were detected in only one sample each: M46L and I85V (protease); K65R, L74I, K219E/R, and K101E (reverse transcriptase) (Figure 4). Eighteen samples (69%) only had one resistance mutation, four samples had two resistance mutations, and four samples had three or more resistance mutations. Twenty samples had mutations from a single class, while the remaining six had dual-class resistance mutations. No samples harbored resistance to all three drug classes (NRTI, NNRTI, PI) (Table 2). Of the three drug classes, resistance to NNRTI class of HIV inhibitors was most frequent. All participants were infected with subtype C HIV-1, except one, who was infected with subtype A.

**Table 2:** Major Drug Resistance Mutation Profiles Detected.

Number ofParticipants	Resistance Profile		
	NRTI	NNRTI	PI
1	K219R	–	–
1	K219E	–	–
14	–	K103N	–
1	–	V106M	–
1	–	–	M46L
1	–	K103N, Y181C	–
1	–	V106M, G190A	–
1	K65R	Y181C	–
1	M184V	K103N	–
1	M184V	K101E, K103N	–
1	M184V	K103N, V106M	–
1	L74I, M184V	K103N	–
1	–	K101E, V106M, G190A	I85V

## Discussion

MTN-009 is the first study to evaluate the prevalence of HIV infection and the proportion of drug-resistant infection in a population of women screening for participation in an ARV-based HIV prevention trial. Several recent surveys conducted in different regions of South Africa among diverse populations have reported that the prevalence of HIV infection among women aged 14–35 years ranges from 22% in the Mbekweni district to as high as 59.3% amongst urban women in the Hlabisa district [22]–[24]. The prevalence of HIV in MTN-009 was 37%, consistent with other recent reports, but higher than anticipated in women interested in participating in HIV prevention trials.

The high HIV prevalence in a prevention trial screening population suggests several possibilities: 1) many women in KwaZulu-Natal still do not know their HIV status; 2) many women who have had an HIV test may want confirmation of their status; 3) many women may be reluctant to report their status because of stigma associated with being HIV positive and/or; 4) HIV positive women may present for screening to HIV prevention studies because they are seeking ART or access to better health care and hope for referrals. Indeed, 39% of women in MTN-009 had CD4+ T cell counts qualifying them for ART (<350 cell/mm^3^), and 12.5% women had CD4 counts low enough to increase risk of AIDS (<200 cells/mm^3^), suggesting either a long duration of unrecognized infection or denial of or unwillingness to report one’s HIV status.

The continued high prevalence of HIV combined with unreliable self-reports of HIV status, particularly in a population targeted for HIV prevention trials, highlights both the need for an effective prevention agent and the importance of improved HIV testing before ARV-based prevention products are disseminated for widespread use. Modeling studies show that the greatest determinant of drug resistance prevalence arising from PrEP is from use of oral PrEP by previously-infected individuals [6].

However, the MTN-009 study aimed to avoid enrolling women who already knew their HIV status. Inclusion criteria involved asking a potential participant about her desire for prevention trial participation. Women were not directly recruited for this study, but rather, some women who presented at sites to screen for a Phase 2B prevention trial evaluating safety and efficacy of tenofovir-based oral tablets or vaginal gel (VOICE) were offered participation in MTN-009. By not advertising MTN-009 in the community, we aimed to avoid enrollment of women who simply wanted a drug resistance test, which is not widely available as part of routine clinical management of HIV positive patients. Although some women may have enrolled into MTN-009 for the benefit of receiving HIV monitoring test results including CD4, HIV RNA or drug resistance, we believe the majority of women who were HIV positive did not knowingly enroll as such.

An unexpectedly high proportion of HIV positive women (35/400 or 8.8%) who were presumed to be untreated had undetectable HIV-1 RNA levels (<40 copies/ml). CD4+ T counts for these participants ranged from 121–1141 cells/mm^3^. An additional 12 participants had detectable RNA levels too low to perform resistance testing (<200 copies/ml). Elite control of HIV infection, which is the spontaneous suppression of plasma viremia with maintenance of high CD4+ T cell levels in the absence of use of antiretroviral agents, occurs in less than 1% of HIV-infected individuals [25]. Consequently, elite control is unlikely to explain the high proportion of participants with very low or undetectable viremia. Knowledge of treatment history, plasma drug levels and long-term follow up of MTN-009 participants is needed to determine if the cause of frequent undetectable HIV-1 RNA is due to antiretroviral use by some participants. Because CD4+ T cells counts are more widely used than HIV RNA in the public sector for monitoring patients on therapy, it is possible that some participants who were already taking ART may have joined the study because they were interested in receiving both an HIV RNA and a CD4 T cell count results to assess their clinical status.

Of the 352 participants with HIV genotypes, 26 (7.4%) were found to have at least one NRTI or NNRTI resistance mutation as identified from the World Health Organization (WHO) list of transmitted resistance [19]. This level of resistance is greater than the <5% range for South Africa first classified in 2008 using the WHO drug resistance threshold survey method, and recently upheld in an analysis of 72 seroconverter sequences from Africa Centre’s 2010 surveillance round [15], [26]. Manasa *et al.* concluded that there is no transmitted drug resistance in KwaZulu-Natal [15]. However our findings verify results from Hunt *et al*. who determined that transmitted resistance in the Gauteng province remains low at <5%, but NNRTI resistance is increasing in KwaZulu-Natal, elevating the threshold classification from low to moderate (5–15%) for that region [17], [27].

Indeed, the most commonly occurring mutation in our study was the NNRTI mutation K103N, which was the only mutation present in virus from 14 women; an additional 5 women had HIV with K103N and other NRTI or NNRTI mutations. Possible sources of K103N acquisition include transmission from a partner on first line therapy in South Africa which includes nevirapine or efavirenz, or selection of resistance from prior exposure to nevirapine for prevention of mother to child transmission (PMTCT) [28]. In a survey of 882 post-partum mothers in antenatal wards in KwaZulu-Natal, 98.6% women reported receiving antenatal care, but only 42.1% of the 312 mothers found to be HIV positive received follow-up care [29]. It is possible that some women in MTN-009 could have been provided single dose nevirapine for prevention of MTCT during delivery in a facility but were not clear on the purpose. From the current data, the origin of NNRTI resistance cannot be determined.

Some participants (5/26, 19%) had resistance profiles that included one or more mutations from both the NRTI and NNRTI classes (Table 2) suggesting that these women may have at one point been on ART or had transmitted drug resistance from a partner on incompletely suppressive ART. Misclassification of ARV treatment status could overestimate resistance prevalence in cross-sectional cohorts [30]. This study relied on participant self-report and could not objectively verify self-knowledge of participant HIV status or prior treatment history to accurately classify resistance observed as transmitted or selected.

Although we cannot be certain that resistance identified in this population was transmitted, we did ask all women via ACASI if she had even been prescribed ARVs and 97% of participants said that they had not. This does not represent an extensive drug history, but it was a confidential one in which the data were collected using a method that was thought to maximize reporting of information that would be less likely to be obtained through a face-to-face interview with a clinician [31], [32]. We do allow for the possibility that some women took ARVs unknowingly, possibly during labor and delivery for the purpose of prevention of transmission to an infant, if they were tested during labor and not clearly informed of the result of their HIV test or did not understand the result during labor and delivery.

This study has some limitations. Because of the cross-sectional design, follow-up of HIV positive participants could not be done to determine whether resistance persisted or to ensure that participants with low CD4 counts or with ARV resistance subsequently accessed HIV treatment and care. Much effort was undertaken to actively follow-up referrals given by the study clinician. Although clinic staff conducted three telephone calls and one home visit, all participants could not be reached to assess the outcome of the referral. Secondly, the study population was enrolled between 2010–2011, just after the changes in the South African national treatment guidelines in 2010 introducing tenofovir as part of first line therapy. We expect that tenofovir may not have been used extensively enough as treatment for HIV infected individuals during the period of participant enrollment to influence the study results. This may explain why the frequency of resistance with K65R was found to be low (<1%) in this study. The frequency of K65R may change over time with increased tenofovir use for first-line HIV treatment. Several studies have indicated that K65R may be more readily selected in subtype C virus, or may be polymorphic at low frequencies [33].

Overall, MTN-009 provides important data on the background of resistance in the setting in which an ARV-based product may eventually be widely disseminated for prevention use. With the increase in ARV use both for treatment and prevention, it will be critical to continue monitoring drug resistance both in clinical trial settings in seroconverters from ARV-based prevention trials, and also in community hospitals and clinics in treatment-naïve and experienced individuals. The dominance of NNRTI resistance in the prevention trial screening population suggests that current ARV-based prevention strategies that include tenofovir will continue to be active in this cohort, but studies evaluating first line treatment failures for cross-resistance with other NNRTI under consideration for use as ARV-based prevention are a high priority.

In addition, this study enables a better understanding of the population of women interested in screening for HIV prevention trials, and women interesting in accessing ARV-based HIV prevention products, which at this point, is only available through a trial setting. By evaluating the prevalence of drug resistant virus in this population, we will be able to better understand the challenges that could be faced when implementing a successful ART-based prevention product. In roll-out of such a product, it is possible that women who know their status, are already on ART, or were previously exposed to ARVs unknowingly would still seek access to HIV prevention. Individuals already harboring drug resistant virus may have additional risk in using ARV-based products meant for prevention.

Further, there are implications for HIV treatment if the prevalence of resistance is increasing in the population. To preserve the effectiveness of tenofovir as first-line therapy, it will be important to continue surveillance in populations where HIV incidence is high and where treatment and prevention roll-outs may be occurring simultaneously.

Finally, this study emphasizes the need for frequent and routine HIV counseling and testing, particularly in settings of high HIV prevalence. ARV-based prevention has great potential to reduce the burden of the HIV epidemic, and the best way to minimize resistance with ARV-based prevention products is avoid its use in HIV-infected individuals [6].
