# Correction: Prevalence of HIV-1 Drug Resistance among Women Screening for HIV Prevention Trials in KwaZulu-Natal, South Africa (MTN-009)

**DOI:** 10.1371/annotation/8096d24f-06de-4e42-8834-b5d3e116253c

**Published:** 2014-01-06

**Authors:** Urvi M. Parikh, Photini Kiepiela, Shayhana Ganesh, Kailazarid Gomez, Stephanie Horn, Krista Eskay, Cliff Kelly, Barbara Mensch, Pamina Gorbach, Lydia Soto-Torres, Gita Ramjee, John W. Mellors, on behalf of the IPTc Taskforce

An error was introduced into the author list during the preparation of this article for publication. The group listed in the last line of the author list on the article page and PDF should read "on behalf of the MTN-009 Protocol Team."

